# How do Rural Second Homes Affect Human Health and Well-being? Review of Potential Impacts

**DOI:** 10.3390/ijerph17186748

**Published:** 2020-09-16

**Authors:** Kati Pitkänen, Jenni Lehtimäki, Riikka Puhakka

**Affiliations:** 1 Environmental Policy Centre, Finnish Environment Institute, PO Box 111, FIN-80101 Joensuu, Finland; kati.pitkanen@environment.fi; 2 Environmental Policy Centre, Finnish Environment Institute, Latokartanonkaari 11, FIN-00790 Helsinki, Finland; jenni.lehtimaki@environment.fi; 3 Ecosystems and Environment Research Programme, Faculty of Biological and Environmental Sciences, University of Helsinki, Niemenkatu 73, FIN-15140 Lahti, Finland

**Keywords:** second home, cottage, outdoor recreation, nature, well-being, health, rural areas

## Abstract

Contact with nature is associated with numerous psychological, physiological and social health and well-being benefits. Outdoor recreation, such as rural second home tourism, provides extensive exposure to the natural environment, but research around health impacts of this exposure is scattered. We review current research on health and well-being impacts of nature and discuss how the characteristics of rural second home environments and their use and users can affect these potential impacts in Finland. We discover four key issues affecting the impacts. First, health and well-being impacts depend on the users; urban people can especially benefit from rural second homes, while child development and the performance of elderly people can also be supported by contact with nature at second homes. Second, the regularity, length and season of second home visits influence the potential to receive benefits as they have an impact on the intensity of nature exposure. Third, the type and quality of second home environment affect contact with nature, such as exposure to health-supporting environmental microbes. Fourth, practices, motives and meanings modify activities and attachment and crucially affect both physical and mental well-being. We conclude that rural second homes have extensive potential to provide nature-related health and well-being benefits and further research is needed.

## 1. Introduction

Research on the health and well-being benefits of nature has increased tremendously during recent years. A variety of positive impacts of the exposure to nature has been shown, ranging from psychological and cognitive benefits to physiological and social benefits [1,2]. This has also inspired researchers of outdoor recreation and leisure to begin to explore the health and well-being benefits of different outdoor activities. The focus has largely been on the health and well-being impacts of recreation in urban parks and green spaces [3]. The benefits of outdoor recreation in more remote and rural locations have been studied in protected areas, for example [4].

Rural second home tourism, that is owning and visiting a secondary residence for leisure purposes in a rural area, is a popular form of nature-based recreation in many countries all over the world. Rural second homes are especially popular in Northern Europe as well as in Russia, where a high share of the urban population has access to a rural second home. Finland has one of the highest numbers of second homes per capita in the world [5]. With a population of 5.5 million people, there are over 500,000 second homes in Finland, and it has been estimated that over 40% of Finns regularly visit one [6,7]. Over 90% of the second homes are located in rural and natural environments [7], and spending time at a second home is considered part of outdoor recreation in Finland [8].

In studies on second homes, the positive impact on people’s health and well-being is usually connected to physical and psychological rejuvenation [9,10,11]. Second homes, in their more rural and natural surroundings, provide a place to escape from everyday life in urban areas [12]. Second homes have also been seen as a part of people’s pursuit for the “good life”, thus being a life strategy that combines the elements of different living environments and mobility in-between of them in order to enhance their owners’ health and well-being [13].

In their review on the health, well-being and second homes nexus in Nordic countries, Åkerlund et al. [10] state that although studies often refer to the positive impact of second homes on people’s well-being, not so much is known about the factors that are behind the positive impacts. For example, although access to nature at second homes is usually highlighted, it has not been elaborated how and why it is important. This paper aims to meet this research gap by exploring the potential health and well-being impacts of rural second homes. We will combine recent knowledge of the health and well-being impacts of nature with studies on second homes, their use and users and second home environments by using Finland as an example. We will explore what kind of exposure to and relationship with nature rural second homes provide and what the potential health and well-being benefits are.

The paper begins with an overview of current research on the health and well-being impacts of second homes and the natural environment. Next, by using the example of second homes in Finland, we will discuss what kind of health and well-being benefits are likely achieved by different second home users at different types of second homes. Finally, the paper will summarize the socio-environmental factors affecting health and well-being benefits of rural second homes.

## 2. Current Knowledge about Health Benefits of Second Homes

### 2.1. Health and Second Homes

In tourism and second home research, a common way to define second homes is as “non-mobile properties owned or rented on a long lease as the occasional residence of a household that usually lives elsewhere” [14,15]. Hence, second homes are understood as being non-mobile buildings located in a certain place and environment. Typically, second homes are located in amenity-rich rural landscapes characterized by an attractive natural environment [15]. In contrast to other types of tourism, second homes are owned or leased for a long time, which implies that second home tourists tend to return to the same place year after year, leading to the formation of a strong place attachment [16,17]. However, despite the long-term place bonding, second home use is occasional and temporary. Second homes are typically used during holiday seasons for recreational purposes [5]. Finally, second homes necessitate a first home, a place where the second homeowners and users live permanently. Rural second homes are often owned by people living permanently in urban areas [18].

Although the positive impacts on well-being are often the motivation for second home tourism, few studies have investigated the health and well-being impacts of rural second homes. To search for literature, we used second homes (synonyms: residential tourism, recreational home, holiday home, vacation home, summer home, summer house, leisure home, cottage, cabin) and health/well-being as the key search terms in the Web of Science.

Based on previous studies, second homes may have a positive impact especially on the health of the elderly. Swedish studies using longitudinal register data showed that owning a second home lowered the odds of early retirement for health reasons [19] and of early death especially among men [20]. In Austria, some elderly people were found to prefer to escape urban areas to more rural second homes for health reasons during hot periods [21]. Moreover, in Poland, the elderly were shown to prefer visiting garden allotments or holiday homes over other types of sport or physical activity [22]. In Russia, working at a second home was found to be important for the physical activity of the elderly [23]. In New Zealand, Walters [11] found that architectural design elements can enhance both physical and psychological well-being at second homes. In a Norwegian study [24], cabin life was indicated to provide a renewal of depleted psychological resources. Interestingly, owning a second home to be able to experience nature correlated with positive emotions and owning it for economic reasons correlated with negative emotions.

Accordingly, some studies question the presumption of the positive impact of second home ownership. Lundmark and Marjavaara [25] suggest that owning a second home can also become a mental burden due to health problems induced by high age, the financial burden of owning multiple residences, or “being stuck” with an inherited second home property. Moreover, second home tourists are a potential risk group for certain environmental or pollution-related health risks. These include contamination of drinking water due to challenging conditions for grey water treatment [26], Lyme disease transmitted by ticks in popular second home areas [27] and second home holidaying being associated with increased risk of exposure to *Legionella* bacteria [28].

Previous studies suggest that the positive impacts of second homes on health are probably due to access to nature and restorative activities enabled by the second home ownership as a counterbalance to urban life [19,20,24]. Therefore, it can be suggested that a green environment instead of just an escape from the city and a relaxed lifestyle is important for health and well-being benefits at second homes.

### 2.2. Health and Well-being Impacts of Nature

Interaction with nature has been shown to promote adults’ and children’s psychological, physiological and social well-being and health in numerous ways [1,2,29]. Direct benefits arise from the opportunity to observe nature and be in a natural environment, and indirect benefits are produced when natural settings encourage physical activity [30]. Interaction with nature increases self-esteem and mood [31,32], reduces feelings of anger and anxiety [33] and has positive effects on emotions and behaviour [34]. Natural areas have been found to be restorative and contribute to attentional recovery and the reduction of mental fatigue [34,35,36]. Hence, exposure to nature has positive effects on concentration, academic performance and the ability to perform mentally challenging tasks [35,37].

Contact with nature alleviates the negative effects of various stressors in urban environments and produces positive changes in human physiology, such as a reduction in blood pressure or heart rate [36,37,38]. Exposure to greenness is also associated with benefits to the immune system, which can reduce the risks of asthma and atopic diseases, for example [39]. Finally, the natural environment and shared nature experiences provide an opportunity for social interaction and strengthening of bonds within families and communities. Nature may foster personal and community identity formation, social activity and social participation [1,31]. Despite the benefits of being in contact with nature, previous studies have also found natural settings to be uncomfortable, distressing or threatening for some people [40].

## 3. Characteristics of Second Homes and Potential Health Benefits

In the following sections, we discuss how the characteristics of rural second homes and users may influence the health and well-being benefits achieved during visits. Based on the definition of second homes (see Section 2.1), we will discuss the health and well-being benefits of second homes in relation to the typical characteristics of the users, patterns of use, second home environments and properties, as well as the motives, activities and practices of second home living in Finland.

### 3.1. Who Receives Health Benefits Provided by Second Homes?

In Finland, second homes are typically *owned* by middle-aged or elderly people. The average age of second homeowners is 63 years, while only 6% of all owners are under 40 years old. The second homeowners are typically couples, with grown-up children living elsewhere, and only 12% of owner households have children under 18 years living at home. Meanwhile, only 16% of second homes are owned by people living alone [6].

Typical health issues in Finnish elderly people include cardiovascular diseases, type 2 diabetes, Alzheimer’s disease as well as depression and musculoskeletal diseases [41]. The risk of most of these diseases can be alleviated with regular physical activity [42], which can be achieved with common daily activities at second homes.

Second home *users* are a much wider group than the owners. In Finland, each of the over 500,000 second homes is annually used by four people on average. Hence, it has been estimated that 2.4 million Finns regularly use second homes (44% of the population) [43]. Access to (i.e., ownership or having the possibility to use) second homes is relatively evenly distributed between different age groups. Hence, the potential health and well-being benefits are received by a large group of people.

Instead of owning, younger households with children more frequently use second homes owned by their parents, relatives or friends [5]. Rural second homes are places where time is spent with close family members or relatives, which enables social interaction and strengthening of social bonds [15]. Interestingly, Cameron-Faulkner et al. [44] found out that communication between parents and their little children was much more responsive and connected in the natural environment than in an indoor environment. Providing possibilities for intergenerational leisure time with children and grandchildren, second homes can support the sense of purpose of elder people, which has been associated with long life [45]. As nature-related skills and knowledge, such as recognizing animals and plants or building a campfire, are often transmitted from one generation to another at second homes, they provide continuity across generations [46].

In Finland, residents of urban areas, particularly of big cities, use second homes more often than rural dwellers [5,18]. When building density increases or access to a private garden is reduced, the use of second homes increases [47]. This indicates that urban people are willing and able to travel to natural settings and thus can derive benefits from green environments missing in their urban home environments. Accordingly, a second home has been described as a gateway to nature for urban people [48].

In particular, the younger generations’ changing relationships with nature have been discussed in Finland as the ability to connect with nature in everyday life has diminished due to urbanization [49]. According to the National Outdoor Recreation Demand Inventory [8], 15- to 24-year-old Finns’ participation in outdoor recreation close to home decreased, but spending time at a second home increased, between 2000 and 2010. This generation was also found to be eager to get a second home in the future [50]. A rural second home is one of the places that enhances Finnish adolescents’ nature-based well-being [49].

Despite the large user groups, socio-economic positions clearly affect access to second homes. Although owning a summer cottage has traditionally had very few elitist connotations in Finland [15], in general the use and ownership of a second home increases with a higher level of education and income [5]. Hence, the health and well-being benefits of second homes are not distributed evenly as people with a higher socio-economic situation are more likely to receive them.

### 3.2. How much Time Spent at Second Homes Produces Health Benefits?

In Finland, rural second homes are used intensively and for relatively long periods annually. The use of second homes has increased slightly over recent years, especially during the autumn, winter and spring seasons [43], which means that potential benefits to health and well-being are increasingly received throughout the year.

A typical Finnish user visits a second home on average 24 times a year, and the median is 13 visits a year. On average, users spend 48 nights at their second home, with a median of 30 nights, indicating a large variation in time spent at second homes. Every fourth second home user spends over 70 nights at a second home and 5% over 150 nights [5]. The average number of visits and the amount of time spent at second homes are high compared with many other forms of outdoor recreation in Finland [8]. Therefore, second home visits have a large potential to increase contact with nature, especially among urban second home users.

**Figure 1:**
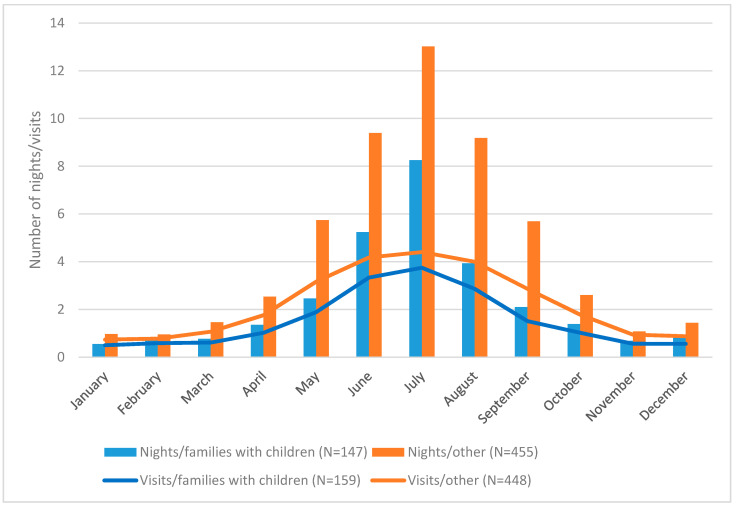
Second home use patterns. Monthly mean values of nights spent at, and number of all visits (overnight and day visits) to, a second home among second home users. Comparison of users that live in a household with children and other types of household (source of data [5]).

Second home use patterns are strongly related to the users’ age, stage of life and permanent living environment. Those second home users under 30 years of age spend the least amount of time at second homes (<20 days a year), after which the number of days increases gradually, reaching a peak after retirement (over 50 days a year) [50]. The youngest and oldest age groups deviate from this pattern. Children who still live with their parents spend more time at second homes than young adults, and those over 80 years spend less time at second homes than other retirees due to difficulties brought by old age [50]. Adults with children tend to spend less time at second homes on average than others (Figure 1).

The location of the second home in relation to the users’ permanent residence has an impact on the frequency and length of second home holidaying. Within a maximum 250–400 km distance from the first home, people often make shorter one-day or weekend trips to their second homes. If the distance is more than 250–400 km, people visit less often, but the visits are usually longer (up to several weeks) [51].

In any age groups, the amount of health and well-being benefits is probably strongly correlated with the time spent at second homes. A recent study in England [52] showed that spending at least 120 min a week in nature—no matter how achieved—is associated with good self-reported health and well-being. In Rogerson et al.’s [53] study, relatively extensive exposure to green environments (12–26 weeks for 2 h a week) was needed to produce long-lasting mental health benefits for vulnerable groups. According to a Finnish study [54], mood is improved when urban green areas are used at least 5 h a month or rural green areas are visited 2–3 times a month. A Finnish study conducted in protected areas [4] indicated that length of stay, and especially spending the night in a nature area, significantly increases the perceived well-being benefits. Time spent at second homes on average exceeds the threshold values for health benefits presented in these previous studies, especially among urban elderly people. However, some health benefits may be achieved much better after “heavy use”. For example, exposure to greenness in the living environment has been found to be protective against adverse mental health outcomes, cardiovascular disease and mortality [30].

However, received health and well-being benefits are influenced by a clear seasonal pattern in the use of rural second homes. The summer vacation season, especially July, is the peak period in terms of the number of visits and time spent at second homes in Finland (Figure 1). The number of nights decreases sharply during spring and autumn. Half of second home users do not spend a night in their second home between October and April; in winter, most of the country is covered by snow at least occasionally. Visits to second homes are also longer during the summer season. Outside the summer season, users make one-day or weekend visits to their second homes [5]. Hence, health and well-being benefits are not evenly received throughout the year at second homes.

### 3.3. What Kind of Second Homes and Environments Produce Health Benefits?

In Finland, second homes are usually located in green or blue environments; 70% are located in a forest and 65% on a seashore, lakeside or riverbank (multiple choices possible). Two-thirds of second homes are located in a scattered settlement [5]. Previous studies have emphasized that an unpolluted environment and a “clean” nature are considered important at second homes [55]. Of landscape features, a location close to clean and good quality water bodies (with access to a private beach) is preferred along unbuilt environments and idyllic rural landscapes [56,57].

The quality of the natural environment surrounding the second home can affect the received health and well-being benefits. Previous studies have shown, for example, that favourite natural-like environments (e.g., woodlands, waterside environments) are found to be more restorative than urban parks [58] and the rural forest more restorative than the same age-class urban forest [59]. The positive contribution of the forest environment to health and well-being has been observed in many studies [60]. Based on the meta-analysis of Barton and Pretty [32], every green environment improved self-esteem and mood, but spending time near a waterside (e.g., beach or river) or participating in water-based activities generated greater effects. An increased diversity of ecosystems has also been associated with a decreased risk of asthma in children [61] and an increased number of rare yard plants with a decreased risk of allergic sensitization in teenagers [62].

Second homes located in rural areas outside cities also have health benefits through the possibility of avoiding air, light and noise pollution. For example, disruption of the natural 24-h day–night cycle caused by artificial light in urban areas may be related to elevated risk of breast or prostate cancer, obesity, depression, diabetes and sleep disorders [63,64]. Similarly, noise pollution from transport, industry and neighbours has been connected with different physical, psychological and cognitive problems [65]. Moreover, air pollution is one of the biggest global health risks associated with numerous non-communicable conditions [66].

Moreover, animal contact at second homes may be important for health and well-being. Encounters with wildlife provide authentic nature experiences for second home users, and in particular, encounters with large animals are cherished [17]. Intentional interactions with nature, such as watching wildlife, have been suggested to increase psychological well-being [67]. Bringing household pets, especially dogs, to the second home can increase the beneficial exposure to microbial species, as pets effectively carry microbes from outdoors to indoors [68].

On the other hand, second home visits may also contain health risks and disbenefits related to the natural environment and animals. Encounters with large animals, such as bears tempted by second homes’ garbage containers, may feel dangerous [69]. Large animals, however, rarely pose a risk to humans in Finland, but potential risks are related to smaller creatures such as venomous vipers, or bees and wasps. Mosquitoes and horseflies are also a common nuisance at second homes. The popular second home areas in Finland coincide with areas of ticks, which can be infected by the *Borrelia* bacterium which causes Lyme disease. Bank voles, often found residing in wood piles near second homes, transmit the Puumala virus, which causes haemorrhagic fever with renal syndrome (epidemic nephropathy). Moreover, exposure to blue-green algae, i.e., cyanobacteria, often found in lake and seawaters in Finland can cause various stomach and skin symptoms.

The health effects can also be related to the type of, and facilities at the, second home. In Finland, second homes are relatively small as their average size is 49 m^2^. However, the size and standard of equipment have increased during recent decades; second homes built in the 2010s were on average 71 m^2^ [6]. Only a third (34%) of second homes are suitable for winter use. However, most second homes (91%) have electricity. A fifth (21%) of second homes has an indoor flush toilet and a quarter (26%) a shower. Most second homes have a fridge (93%) but, for example, a dishwasher is less common (15%) [43]. The increasing standard of equipment increases the possibilities of using second homes longer, especially outside the warm months, enhancing the opportunities for receiving health benefits. However, the standard of equipment at an average second home is not as high as at permanent homes, which may involve some health risks. For example, hygienic conditions for preparing and preserving food may be lacking.

Similarly, the quality of drinking water can greatly influence health benefits received at second homes. Half of Finnish second homes bring water from elsewhere in canisters, a third has an own well, 17% are connected to municipal or local water infrastructure and at every tenth second home drinking water is taken directly from a lake, river, spring or the sea [43]. Normal municipal tap water in Finnish cities is clean from any microbial exposure. Even though clean water is globally a major health definer, some studies consider that too clean water in high-income countries can also have a negative impact on the immune system when exposure to potentially beneficial microbial contaminations is missing [70]. Moreover, washing dishes by hand has been associated with a reduced risk of allergic disease development among children [71]. However, the use of unpurified drinking water or contamination of water supplies may also become a health risk at second homes, such as Norovirus or Campylobacter outbreaks.

### 3.4. How do Motives, Practices and Activities at Second Homes Affect Health Benefits?

One of the main motives for second home holidaying in Finland is getting close to nature and a peaceful environment [15]. Second homes represent a simpler and more natural and ecological way of life. At one end are second home users who seek a temporary and recreational escape from their everyday (urban) environment to a more peaceful and natural environment. At the other end are those for whom second homes provide possibilities for creative work and self-fulfilment not possible at the permanent home [12]. These motives are important especially for men (see [72]).

The natural environment plays an important role in the creation of second home users’ attachment to place, that is the affective and emotional bonds people have with places [17]. In Finland, second homes often have a connection to family history and roots, providing a sense of continuity in the modern world [56]. Thus, they may be considered emotionally more significant places than the official first homes [10,15]. The way people sense place influences the way people interact with place; a strong sense of place is usually positively related to self-perceived mental health [73].

Nature is present in many popular activities at second homes. At Finnish second homes, time is spent engaging in various recreational activities such as observing and admiring nature and the landscape, swimming, rowing, boating, going for walks or hikes, barbecuing, having a sauna and just relaxing in and outside the cottage. In addition, more use-oriented consumptive activities such as fishing, picking berries and mushrooms, forest work, gardening, chopping firewood, building and renovating and heating the sauna, as well as cleaning, cooking and preparing meals in and outside the second home are popular [17,56]. In general, access to a rural second home was found to increase the rate of participation in outdoor recreation and consumptive activities in all age groups in Finland [8,48].

Many of the activities at second homes include close physical contact with water, plants, soil and hence environmental microbes. Environmental conditions and microbiota (i.e., a microbial community in a defined ecosystem) tend to shape the composition of human microbiota, especially on skin and in airways, but also in the gut [74,75,76,77,78,79]. Thus, environmental microbial richness can influence all parts of human microbiota, which again closely interacts with the immune system [80]. Indeed, changes in human microbiota or microbial exposure have been associated with the alleviation of the risk of numerous immune-related diseases such as asthma and atopic diseases, Alzheimer’s disease, and mental illnesses such as depression [81,82,83,84].

Moreover, other environmental exposures can benefit human health. In the study of Lynch et al. [85], children who were exposed not just to environmental microbiota but also to a diverse set of allergens (molecules which the immune system recognizes, such as pollens) were less likely to develop allergic sensitization than those children exposed to only either one. Therefore, the natural environment can provide multiple stimulating properties which support health. However, for people suffering from severe allergies or hypersensitivities, increased exposure to allergens and microbes at second homes can produce adverse health effects.

Since second home use concentrates on the summer season, winter activities are less frequent. In a Norwegian study [24], winter use (ski tours in particular) was found to be positively correlated with general subjective well-being and positive emotions experienced at the second home. However, it is unclear whether microbial exposure is health-promoting also during winter months as there is seasonal variation in microbial exposure. The relative abundances of several bacterial taxa associated with beneficial health effects were shown to be lower at study subjects’ homes in winter compared with summer, indicating limited exposure during winter months [86].

Besides what people do, also what people eat at second homes has potential health effects. Studies on food consumption have shown that Finnish second home users appreciate local products, and most bakery products, farmed berries, potatoes and vegetables consumed at second homes are locally produced [87,88]. In addition, people pick berries and mushrooms, fish and grow vegetables and fruits in their own second home gardens. Second home users seem to prefer relatively healthy diets with lots of vegetables, fruits and berries, although barbecuing sausages and red meat is also common and can form carcinogens. Getting food directly from forests, lakes, a person’s own garden or farms can have an additional positive impact by providing physical activity and beneficial microbial exposure. It has been found out, for example, that the risk of allergic disease development among children was reduced if the family bought food directly from farms [71].

## 4. Discussion

Based on our review, rural second homes have a high potential to provide health and well-being benefits associated with an increased connection with nature. Hartig et al. [2] and Tyrväinen et al. [89] have summarized that contact with nature may affect health via multiple pathways. The identified pathways are (1) decreased negative environmental exposures such as pollution and noise, (2) decreased stress and hence increased restoration, (3) increased physical activity, (4) increased social contact or personal time and space, and (5) a strengthened or balanced immune response. These pathways are influenced by the type, quality and amount of the natural environment, the frequency and duration of contact, as well as the activities performed in nature. The strength and focus of the positive effects are subject to modification by individual or contextual variables (e.g., age, gender, socio-economic status, health status, societal/cultural context and environmental preferences) [2,89]. We expect these features to also contribute to the health and well-being of second home users. To complement these findings, based on our review, we propose that the health and well-being benefits provided by second homes also arise from the role of nature in enhancing attachment to place, possibilities for self-realization and finding a sense of meaning and continuity. In terms of effect modification, our review highlights the importance of previous experiences, memories and length of the personal relationship with the place.

In Finland, second home use is widespread in society with a large part of the population having regular access to a rural second home. Based on our review, the elderly and children living in urban areas can especially benefit from the contact with nature provided by rural second homes. The use of second homes is most intense among the elderly, who often have retired, and second homes may be especially important for the physical and mental health of older males. For them, the active lifestyle and increased contact with nature provided by second homes can increase the number of healthy and active years by alleviating or preventing diseases that tend to accumulate in older age. More widely, second homes can partly replace the missing contact with greenery during holidays and weekends for urban people. Urban children can greatly benefit from visits to rural second homes during their intensive phase of both physical and mental development. A second home may also be important for maintaining a connection with nature during adolescent years which have been described as a “time-out” in people’s preference for the natural world [90].

The population is rapidly ageing and increasingly concentrates in urban areas in Finland. Strandell et al. [18] anticipate that the urbanization and demographic developments will not decrease the popularity of second home use in the future. Hence, it can be expected that second homes will become increasingly important for public health. Moreover, global events can shape their popularity; for example, as a consequence of COVID-19, second home sales and rentals have increased in Finland [91].

Research on health and well-being impacts of outdoor recreation is based on the assumption that regular (e.g., weekly) visits to natural settings maintain the benefits derived from that environment. However, seasonal use of second homes can significantly affect the received benefits. Currently, it is not understood whether health benefits achieved during the peak season (typically summer) last during the low season (winter), even though lowered odds for early retirement for health reasons [19] and decreased mortality [20] among second home owners suggest long-term benefits. Studies focusing on regular outdoor recreation are hard to generalize to second home use, which provides much more intensive but seasonal exposure to nature. Psychological and social benefits such as strengthened social bonds or increased mood and positive emotions arising from contact with nature, good memories and positive place attachment can carry over during the periods second homes are not visited. However, it is likely that physical benefits such as the effects of microbial exposure on human microbiota decrease relatively fast after returning to the (urban) first home [92]. Further research is needed on the long-term health and well-being impacts of second home holidaying in different seasons.

However, second home use is currently changing from seasonal use to year-round use in Finland. The standard of equipment is increasing, and many second homes are suitable for winter use. The increasing standard of equipment in second homes may, on the one hand, lead to an increase in time spent indoors and a decrease in direct contact with nature. On the other hand, better equipped second homes are visited more intensively also during the low seasons, which increases the possibilities of receiving health and well-being benefits throughout the year.

The quality and diversity of green or blue environments around second homes are important determinants of received health and well-being benefits. The popular second home environments such as forests, lakes and rural landscapes have been associated with many benefits in previous studies. Finnish second homes are typically located in the middle of nature, spatially apart from the other community structures, which gives good possibilities to avoid negative exposures to air pollution, noise and artificial light. When nature practically starts at the doorstep of second homes, the likelihood of receiving many nature-related psychological and physiological benefits increases independently of which kind of outdoor activities people engage in. Moreover, the lack of modern conveniences enhances contact with nature, such as environmental microbial exposure, through washing in natural waters and chopping firewood, for example. Personal preferences, practices and activities further shape the strength of contact with nature, influencing health benefits. The differing motives for second home tourism indicate that well-being benefits can be received from different sources. For example, inactive ways of spending time at the second home can relieve stress just like active maintenance projects can. Health and well-being impacts of various second home activities or environments are worth exploring in more detail in future studies. In particular, the quality of second home environments has been neither investigated nor compared with that of urban green spaces or gardens.

Some reservations have to be taken into account when interpreting the results of our review. For example, some health benefits of second homes may also come from decreased exposure to common risk factors of modern life, such as daily chemicals, and not just from increased exposure to a green environment [93]. These inversely correlated features can be hard to disentangle. Our study also reminds us that not all contact with nature benefits health but that there are several health risks related to exposure to harmful bacteria, viruses or allergens. Due to climate change, natural environments are changing, and pathogens are becoming more common, which may have effects on the health benefits of second homes. In urbanizing societies, some may also experience nature at second homes as overwhelming or scary rather than relaxing. As there is little actual research on the health and well-being impacts of second homes, some of our conclusions may be under- or overstatements. Despite these reservations, our study illustrates how outdoor recreation, such as second home tourism, can have a significant impact on public health in urbanizing and ageing societies through providing regular and continuous access to nature. This finding is applicable to other countries with similar second home environments, practices and traditions, and where second home ownership and use is widespread in the society such as the other Nordic countries and Russia [94,95]. More in general, the study illustrates the potential health impacts of rural tourism and outdoor recreation.

## 5. Conclusions

**Figure 2:**
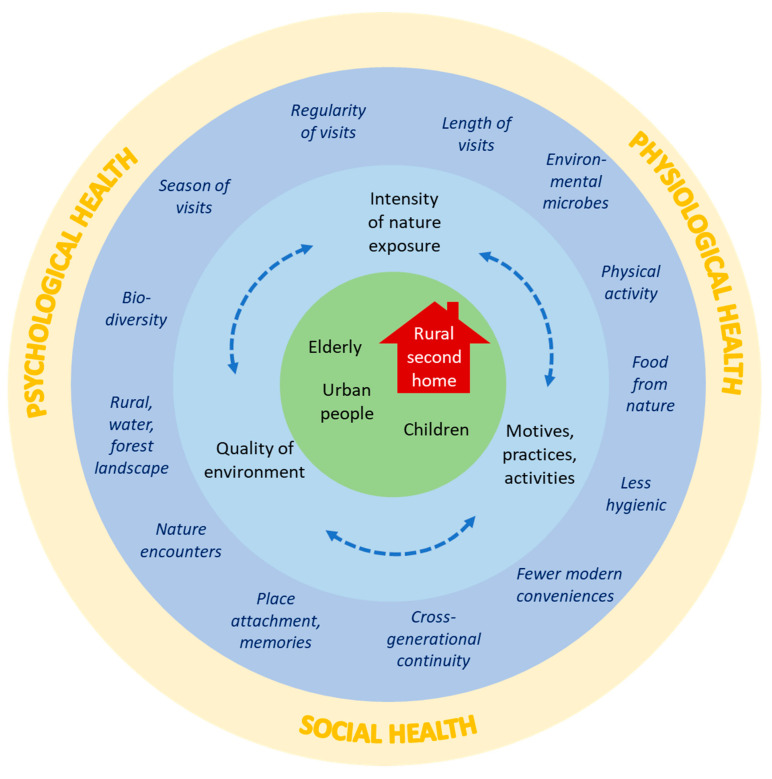
Features and users of second homes and the potential influence of second homes on health and well-being. The inner circle represents a second home and its users, who ultimately influence the potential health benefits. The second circle represents the major features of second homes and their use, which potentially influence health. The third circle details and provides examples about the major features. The outer circle describes the dimensions of human health, which can be influenced by second home use, as well as underlines that all dimensions of health are related.

This review has explored the potential health and well-being impacts of rural second homes. We conclude that contact with nature at second homes can benefit psychological, physiological and social health and well-being in numerous ways. The received benefits affected by the socio-physical environment of second homes are illustrated in Figure 2. We discovered four key issues affecting the health and well-being impacts. First, as illustrated in the inner-most circle in Figure 2, impacts depend on the users; urban people can especially benefit from rural second homes, while also child development and the performance of elderly people can be supported by connection with nature at second homes. Second, the two blue circles illustrate the features and mechanisms of second home stays that influence the health benefits. The regularity, length and season of second home visits influence the long-term health benefits as they impact the intensity of the exposure to nature. The quality of the second home environment in terms of the biodiversity and degree of rurality, for example, affects the contact with nature, such as exposure to health-supporting environmental microbes. Practices, motives and meanings modify activities and attachments and crucially affect both physical and mental health. Finally, the outer yellow circle illustrates the three overlapping and parallel dimensions of health that second home stays have an impact on.

The study emphasises and illustrates the importance of people’s multiple everyday and leisure environments for public health. In modern times, people live increasingly mobile and multi-local lives sharing their everyday lives between multiple locations with very different types of possibilities for contact with nature. Like in our study, urban people’s use of second homes can be important in sustaining their regular access and exposure to more natural and rural environments. However, future changes in the first and second homes’ socio-physical environment and society can have significant consequences for the received health and well-being benefits and should be taken into consideration by planners and policy makers. To support this work, more empirical research is needed to obtain evidence of the health and well-being impacts of second homes.
